# Healthcare Workers’ Knowledge, Attitudes, and Practices about Vaccinations: A Cross-Sectional Study in Italy

**DOI:** 10.3390/vaccines8020148

**Published:** 2020-03-26

**Authors:** Concetta P. Pelullo, Giorgia Della Polla, Francesco Napolitano, Gabriella Di Giuseppe, Italo F. Angelillo

**Affiliations:** Department of Experimental Medicine, University of Campania “Luigi Vanvitelli”, 80138 Naples, Italy; concettapaola.pelullo@unicampania.it (C.P.P.); giorgia.dellapolla@unicampania.it (G.D.P.); francesco.napolitano2@unicampania.it (F.N.); gabriella.digiuseppe@unicampania.it (G.D.G.)

**Keywords:** healthcare workers, Italy, knowledge, recommended vaccinations, survey, vaccination coverage

## Abstract

The cross-sectional study assessed the knowledge, attitudes, and practices regarding the recommended vaccinations and factors affecting such outcomes among a sample of healthcare workers (HCWs) in public hospitals in Italy. Only 14.1% knew all the recommended vaccinations for HCWs. Physicians and those who had received information about vaccinations from scientific journals, educational activities, or professional associations were more likely to have this knowledge, while those aged 36–45 were more likely to have less knowledge than those in the age group below 36 years. Only 57.3% agreed that the information received about vaccinations was reliable. Respondents who had children, who worked in pediatric/neonatal wards, who were more knowledgeable, or who did not need further information about vaccinations considered the available information to be reliable. Only 17.7% of respondents always recommended vaccinations to their patients. This behavior was more likely to occur in physicians, in HCWs, in pediatric/neonatal wards, in those who considered the information received about vaccinations reliable, and in those who considered themselves to be at high risk of transmitting an infectious disease to their patients. Health promotion programs and efforts are needed to improve the level of knowledge about vaccinations and immunization coverage among HCWs.

## 1. Introduction

Healthcare workers (HCWs) have an important role in informing, advising, and promoting vaccinations in accordance with the most up-to-date scientific evidence as a preventive measure for self-protection from the risks of contracting the vaccine-preventable diseases and, consequently, of their transmission to other members of staff and their patients, particularly the vulnerable groups [1,2].

In view of this, in Italy, vaccinations against hepatitis B, measles–mumps–rubella, varicella, pertussis, and influenza are currently highly recommended for HCWs [3], and they are also encouraged to receive tetanus and diphtheria booster doses. However, vaccination coverage for HCWs remains suboptimal. For example, the seasonal influenza coverage was 15.6% in 2017 [4], still falling below the national target of 75%, despite the fact that it can spread in hospital settings and represents a leading cause of healthcare-associated infections. Therefore, there have been renewed calls to improve coverage. Moreover, it is now well established that the low uptake of vaccinations among HCWs may be attributable to many factors. Indeed, HCWs’ knowledge gaps and poor attitudes regarding vaccinations, concerns about safety, level of perceived risk, difficulties in accessing the vaccinations, and communication strategies have been documented to influence the variation in uptake among HCWs with an increased risk of infection and negative impacts on the patients and the community [5,6].

Reports worldwide have been carried out to examine the knowledge, attitudes, and practices of HCWs in relation to vaccinations [7,8,9]. Despite the low vaccination uptake rates in Italy, there have been few previous studies conducted to explore the knowledge, attitudes, and practices of HCWs [10,11,12]. Thus, the primary objective of the current investigation was to assess the knowledge, attitudes, and practices regarding the recommended vaccinations among a large sample of HCWs in Italy. We also aim to understand the factors affecting such outcomes in order to provide evidence supporting the development of a comprehensive vaccinations plan for HCWs.

## 2. Materials and Methods

### 2.1. Study Design and Setting

The cross-sectional study lasted from May to July of 2019 and took place in six randomly selected public general hospitals distributed in Naples, Italy. The study population of HCWs was randomly sampled from the selected hospitals. The sample size of the study was calculated using a single population proportion formula with the assumption of 15% of HCWs who knew all the vaccinations recommended for them, a confidence level of 95%, a margin of error at 5%, and a response rate of 50%. The final sample size of the study was 392 HCWs.

### 2.2. Data Collection

Before commencing the survey, hospital directors were contacted with an introductory letter requesting their collaboration explaining the objectives of the study and the methodology, after which they were asked to provide a list of HCWs. After the approval was received, the random sample of HCWs received an envelope containing the questionnaire. This questionnaire was accompanied by an information sheet that outlined the rationale for the study and explained that participation was voluntary, that they could leave the assessment at any time, and that full confidentiality and anonymity would be maintained. Included were a written informed consent form and an envelope to return the completed questionnaire and the consent form. The completed surveys were anonymous. Participants were given two weeks to complete the survey, and a follow-up visit was made weekly to each hospital to ensure maximum participation of the sampled HCWs. All participants gave written informed consent before participating in the survey. There were no incentives offered to those who participated in the survey.

### 2.3. Survey Questionnaire

A pilot test among 50 HCWs was undertaken in order to evaluate the feasibility of the study design, readability of the items, and reliability of the questionnaire. Data from the pilot were included in the final analysis. The study protocol, questionnaire, and informed consent were reviewed and approved by the Ethics Committee of the Teaching Hospital of the University of Campania ‘‘Luigi Vanvitelli” (number 411). The research team developed a self-administrated structured questionnaire containing five fields of inquiry. In the first part of the survey, there were eight questions, which asked age, gender, marital status, number of children, professional status, length of practice, type of ward, and previous practice in other unit areas. The second part of the survey consisted of 14 predefined questions that asked HCWs about their knowledge regarding the vaccinations recommended for them. The questions could be answered as “yes”, “no”, or “do not know”. The third section consisted of questions about the sources and needs of information regarding vaccines recommended for HCWs. The fourth section contained questions that measured attitudes towards vaccinations for HCWs and their patients. It contained questions surrounding the perception of the risk of contracting a vaccine-preventable infectious disease and of transmitting one to patients, beliefs about the usefulness of the recommended vaccines for HCWs and patients, and reliability of the information received about vaccinations. All questions pertaining to attitudes were measured on a 5-point Likert scale with options from “strongly disagree” to “strongly agree”. The fifth section asked about practices regarding vaccinations. The questions in this section asked whether HCWs recommended vaccinations to their patients, whether they convince their patients to vaccinate, and whether they discussed vaccinations with colleagues. The practice was measured on five-response options from “never” to “always”. Finally, coverage for all recommended vaccines for HCWs was also investigated, and the response options included ‘‘yes”, ‘‘no”, “do not remember”, or “naturally immunized”.

### 2.4. Data Analysis

Stata software version 15 (StataCorp, College Station, TX, USA) [13] was used for analyzing the data obtained from the questionnaires. Descriptive and inferential statistics were used to analyze the data. Descriptive statistics (frequencies, means, standard deviations) have been conducted to describe the characteristics of the study population and the main variables. The bivariate analysis has been conducted using the chi-squared test for the categorical variables and the Student’s t-test for the continuous variables, as appropriate. Those independent variables that attained a significance level of *p*-value less than or equal to 0.25 [14] in the univariate analysis were included in multivariate linear and logistic regression models to identify the factors predicting the following outcomes of interest: HCWs who knew the vaccinations recommended for them (continuous) (Model 1); HCWs who agreed or strongly agreed that the information received about vaccinations was reliable (no = 0; yes = 1) (Model 2); and HCWs who always recommended the vaccinations to their patients (no = 0; yes = 1) (Model 3). To determine the level of knowledge about the nine recommended vaccines for HCWs, a score of “1” was put for the correct answer and “0” for the incorrect or unknown answers. Therefore, the total knowledge score was calculated, and it ranged from 0 to 9.

The following independent variables were included in all models: gender (male = 0; female = 1); age, in years (< 36 = 1; 36–45 = 2; > 45 = 3); marital status (unmarried/separated/divorced/widowed = 0; married = 1); length of practice, in years (continuous); having a child (no = 0; yes = 1); working in pediatric/neonatal wards (no = 0; yes = 1); and use of scientific journals, educational activities, or professional associations as sources of information about the vaccines recommended for the HCWs (no = 0; yes = 1). In Models 2 and 3, the variable “knowledge of the vaccines recommended for HCWs” (continuous) was also included. In Model 3, the variables “considering themselves at high-risk of contracting an infectious disease during their practice” (no = 0; yes = 1), “considering themselves at high-risk of transmitting an infectious disease to their patients” (no = 0; yes = 1), “believing that the information received about vaccinations was reliable” (no = 0; yes = 1), “considering the vaccinations beneficial for their own health” (no = 0; yes = 1), “being favorable to vaccinations” (no = 0; yes = 1), and “considering the vaccinations beneficial for their patients” (no = 0; yes = 1) were also included. A stepwise backward variable selection was applied to test for factors predicting outcomes of interest, and factors with *p*-values <0.2 and >0.4 were included and excluded in the model, respectively. Odds ratios (ORs) and their 95% confidence intervals (CIs) were presented to express the results of the logistic regression analysis and standardized regression coefficients for the linear regression model. For all analyses, the tests for significance were two-sided, and *p*-values of less than 0.05 were considered significant.

## 3. Results

Among the total of 715 questionnaires distributed, 412 HCWs agreed to participate and returned the survey for an overall response rate of 57.6%.

The main socio-demographic and professional characteristics of the study participants are displayed in Table 1. The mean age was 45.8 years (range 23–66), 61.7% were female, 55% were married, 64.6% had a child, the majority (69.8%) were nurses, 16% worked in pediatric/neonatal wards, and the mean length of practice was 10.9 (1–41) years.

Overall, only 14.1% of the respondents were aware of all recommended vaccinations for HCWs. Out of a maximum knowledge score of 9, the mean score achieved by the participants was 4.8 ± 2.8 (SD), and 1.9% did not answer any question correctly. The majority of HCWs correctly identified that the vaccines against hepatitis B (94.7%) and influenza (68%) were recommended for them. In contrast, lower values have been observed for measles (54.6%), tetanus (49.8%), rubella (48.5%), varicella (45.4%), mumps (43.5%), pertussis (42%), and diphtheria (37.6%). Table 2 showed the results of multivariate linear and logistic regression analysis of the effect of predictors on the three different outcomes of interest. Physicians and those who had received information about vaccinations from scientific journals, educational activities, or professional associations were more likely to know all the recommended vaccinations for HCWs. Respondents aged 36–45 years were more likely to have lower knowledge than those in the age group below 36 years (Model 1).

On the importance of vaccinations for HCWs, the vast majority (88%) were favorable and considered the immunizations recommended useful to protect their own health (87.1%) and their patients (86.6%). Only 50.9% and 36.5% of the HCWs considered themselves at high risk of contracting and of transmitting an infectious disease during their activity. Only 57.3% agreed or strongly agreed that the information received about vaccinations was reliable. The multivariate logistic regression model showed that HCWs having a child, those who worked in pediatric/neonatal wards, those more knowledgeable, and those who did not need further information about vaccinations, considered the information received about vaccinations reliable (Model 2 in Table 2). Only 17.7% stated that they always recommended vaccinations to their patients. The multivariate logistic regression model indicated that this behavior was more likely to occur in physicians, in HCWs working in pediatric/neonatal wards, in those who considered the information received about vaccinations reliable, and in those who considered themselves at high risk of transmitting an infectious disease to their patients. HCWs aged 36–45 years were less likely to recommend the vaccinations than those in the age group below 36 years (Model 3 in Table 2). Respectively, 26.9% and 6.2% of HCWs stated that they often/always convinced their patients to vaccinate and always discussed with colleagues who were not favorable for vaccinations. Only 12.4% of HCWs were offered on-site vaccinations at their workplace.

The self-reported coverage for the vaccines recommended for HCWs by the study participants is reported in Table 3. The coverage was high only for hepatitis B (78.6%), whereas, among those susceptible, very low values were reported for tetanus (17%), rubella (13.8%), measles (12.1%), varicella (8.6%), mumps (8.4%), diphtheria (3.6%), and pertussis (2.2%). Only 21.6% had received the influenza vaccine in the last year.

The vast majority of the HCWs stated that they had obtained information about vaccinations (81.5%). The preferred method for acquiring information was the internet (38.6%), followed by educational activities (36.9%), scientific journals (31.3%), colleagues (27.4%), and professional associations (24.7%). Moreover, 58% of HCWs reported the need for additional information about vaccinations.

## 4. Discussion

This study provides novel insights into vaccination knowledge, attitudes, and coverage among a large sample of Italian HCWs.

The results of the present survey showed that the HCWs had a poor level of knowledge, since only 14.1% were aware and knowledgeable about all vaccines recommended for them. It is well known that HCWs have a crucial role in vaccinations, and they should have adequate knowledge in order to correctly inform the population and the most fragile and susceptible patients. Indeed, several recent investigations among different groups of individuals conducted in the same geographic area have underlined that HCWs are the most important and trusted source of information on vaccine-preventable infectious diseases [15,16,17,18]. Therefore, the observed poor level of knowledge is a serious concern because HCWs may underestimate the risks of contracting the vaccine-preventable diseases and, consequently, of their transmission to other members of staff and their patients, particularly those in the vulnerable groups. This signals an urgent need for infection prevention and control education to guard the safety of the HCWs. Furthermore, the results of the multivariate regression analysis showed that a higher level of knowledge and more frequent recommendation of vaccinations to the patients were more likely to be observed in physicians and in the younger age group. Additionally, previous investigations have found that doctors have a higher knowledge of vaccine-related topics than nurses [12,19,20]. This may be due to the fact that more coursework concerning vaccinations has emerged in medical degrees in recent years. Therefore, more work needs to be done to strengthen the nursing training structure and curriculum, enhance the tools to understand the benefits of vaccines, and enable students to contribute to the prevention of infectious diseases, due to their critical future role.

A disturbing result was that only 57.3% agreed or strongly agreed that the information received about vaccinations was reliable. This issue could be addressed through targeted education and awareness campaigns to combat misconceptions and concerns regarding vaccine safety and efficacy and to increase confidence in vaccines.

An objective of this investigation was to evaluate the HCWs’ self-reported vaccine coverage; except for hepatitis B, all rates were very low, including those for influenza (21.6%), tetanus (17%), and measles (12.1%). Overall, the low coverage among this risk group is alarming. Indeed, since it is well established that HCWs play a role in nosocomial transmission of infectious diseases, the low-level coverage of those susceptible did not contribute to limit the spread of infections and might not provide indirect protection for the patients. The findings of previous investigations showed a higher coverage for several vaccinations in different groups of HCWs compared to the results of this survey, with values of 41.7% [21] and 45.7% [22] for influenza in Slovenia and France, respectively; values of 93.7% and 80.5% were reported for diphtheria–tetanus–acellular-pertussis and measles–mumps–rubella in Italy, respectively [12]; values of 88% and 72% were reported for diphtheria–tetanus–poliomyelitis and pertussis in France, respectively [23]; and a value of 50.5% was reported for pertussis in Australia [24]. One possible explanation for the observed poor immunization coverage could be the lack of free on-site vaccines at work. In fact, only 12.4% of the selected hospitals offered the vaccinations on-site and free of charge to HCWs at their workplace. It is essential that all HCWs should be trained on the risks of occupational exposure and on preventive and protective measures, given that their low vaccinations coverage exposes themselves and their patients to the risk of contracting an infectious disease. Therefore, educational campaigns are needed to improve the HCWs’ knowledge about the recommended vaccinations in order to increase their coverage and also about hand hygiene in order to prevent healthcare-associated infections.

Another point of interest was that the frequency of the sampled HCWs who recommended the vaccinations to the patients was very low, as less than one in five declared to make the recommendation. Previous studies conducted in the United States highlighted that 98% of primary care providers recommended influenza vaccine to those ≥ 65 years, 90% to adults 50–64 years, and 75% to 19–49 years [25]; 54.4% and 54.7% of physicians recommended diphtheria–tetanus–acellular-pertussis and zoster vaccines, respectively, to patients ≥ 65 years [26]; and 94.7% of clinicians recommended vaccines to adults [27]. In the present study, HCWs were most likely to feel confident in trusting online information sources to search about vaccinations. The results showed that traditional sources of information for this group, such as scientific journals, educational activities, or professional associations, although significantly associated with a higher level of knowledge, were secondary to internet-based sources of information. HCWs tended to look independently for information rather than ask for it. This is of concern as several studies have found that receiving information from congress/educational courses and scientific journals is a significant driving factor for improving HCWs’ understanding and therefore increasing immunization coverage [7,11,28,29,30,31,32].

As with all similar research, it is important to reflect on some potential limitations of this survey. Firstly, the cross-sectional nature limits inferences on the directional causality of the associations observed in this study. Secondly, the sample was selected only from one geographic area; therefore, the collected responses might not be generalizable to other parts of the country. Thirdly, the information about vaccination status was self-reported and not based on the vaccination records of staff. This might be prone to recall, declaration, or desirability biases; therefore, an over- or underestimation of coverage could occur. However, the fact that the survey maintained full confidentiality and anonymity may suggest that the responses were likely to be authentic, with minimal social desirability bias.

## 5. Conclusions

In conclusion, these findings are valuable for the design and planning of training to ensure that HCWs improve their level of knowledge about vaccinations and develop skills with the resultant positive impact on their responsibility to patients and on their immunization coverage in a geographic area with disturbingly low vaccination implementation.

## Figures and Tables

**Table 1:** Participants’ socio-demographic and professional characteristics.

Characteristics	N	%
*Gender*		
Female	251	61.7
Male	156	38.3
*Age, years*	45.8 ± 9.7 (23–66) *	
< 36	62	15.9
36–45	119	30.4
> 45	210	53.7
*Marital status*		
Married	221	55
Unmarried/separated/divorced/widowed	181	45
*Having a child*		
No	146	35.4
Yes	266	64.6
*Profession*		
Nurses	280	69.8
Physicians	100	25
Nursing supporting staff	21	5.2
*Length of practice, years*	10.9 ± 9.2 (1–41) *	
*Workplace*		
Medical wards	227	55.8
Pediatric/neonatal wards	65	16
Emergency room	59	14.5
Intensive care unit	56	13.7

* Mean ± standard deviation (range); Number for each item may not add up to the total number of study population due to missing values.

**Table 2:** Multivariate linear and logistic regression analyses to characterize factors associated with the different outcomes of interest.

**Model 1: Knowledge about all recommended** **vaccinations for HCWs**	**Coeff.**	**SE**	***t***	***p***
*F*(5, 376) = 7.67; *R*^2^ = 9.3%; adjusted *R*^2^ = 8.1%; *p* < 0.0001				
Physicians	1.21	0.34	3.59	< 0.001
Information from scientific journals, educational activities, or professional associations	0.87	0.3	2.91	0.004
Age (years)				
< 36	1*			
36–45	−1.01	0.46	−2.19	0.029
> 45	−0.76	0.44	−1.71	0.087
Not having children	−0.49	0.32	−1.52	0.129
**Model 2. HCWs who agreed or strongly agreed that the information received about vaccinations was reliable**	**OR**	**SE**	**95% CI**	***p***
Log likelihood = −226.74; χ^2^ = 54.86 (9 *df*); *p* < 0.0001				
Not needing further information about vaccinations	0.32	0.08	0.2–0.51	< 0.001
Working in pediatric/neonatal wards	2.37	0.81	1.21–4.62	0.012
Having a child	1.85	0.54	1.04–3.28	0.036
Having higher knowledge about recommended vaccinations for HCWs	1.09	0.04	1.01–1.18	0.041
Physicians	1.69	0.48	0.96–2.96	0.07
Male	0.73	0.18	0.45–1.19	0.21
Married	1.37	0.38	0.79–2.38	0.26
Information from scientific journals, educational activities, or professional associations	1.29	0.31	0.8–2.09	0.29
Age (years)				
< 36	1*			
36-45	0.78	0.19	0.48-1.26	0.31
**Model 3. HCWs who always recommended the vaccinations to their patients**	**OR**	**SE**	**95% CI**	***p***
Log likelihood = −118.34; χ^2^ = 110.69 (9 *df*); *p* < 0.0001				
Working in pediatric/neonatal wards	4.39	1.71	2.04–9.43	< 0.001
Physicians	6.52	2.37	3.2–13.3	< 0.001
Considering reliable the information received about vaccinations	3.67	1.47	1.67–8.05	0.001
Age (years)				
< 36	1*			
36–45	0.34	0.14	0.15–0.78	0.011
Considering themselves at high risk of transmitting an infectious disease to their patients	2.47	0.97	1.14–5.35	0.022
Being favorable to vaccinations	3.27	2.71	0.64–16.64	0.15
Considering useful the vaccinations for their patients	1.68	0.7	0.74–3.8	0.21
Having higher knowledge about recommended vaccinations for HCWs	1.07	0.06	0.96–1.21	0.22

* Reference category.

**Table 3:** Healthcare workers’ self-reported vaccination coverage.

Vaccination	Vaccinated		Not known		Not Vaccinated	
	N	%	N	%	N	%
Hepatitis B	324	78.6	19	4.6	69	16.8
Influenza	89	21.6	8	1.9	315	76.5
Tetanus	70	17.7	58	14.7	267	67.6
Rubella *	22	13.8	37	23.1	101	63.1
Measles *	14	12.1	72	62	30	25.9
Varicella *	11	8.6	37	28.9	80	62.5
Mumps *	13	8.4	29	18.7	113	72.9
Diphtheria	15	3.6	95	23.1	302	73.3
Pertussis *	5	2.2	78	34.2	145	63.6

* Among those susceptible.
